# Does a Mediterranean-Type Diet Reduce Cancer Risk?

**DOI:** 10.1007/s13668-015-0141-7

**Published:** 2015-09-23

**Authors:** Lukas Schwingshackl, Georg Hoffmann

**Affiliations:** German Institute of Human Nutrition Potsdam-Rehbruecke (DIfE), Arthur-Scheunert-Allee 114-116, 14558 Nuthetal, Germany; Faculty of Life Sciences, Department of Nutritional Sciences, University of Vienna, Althanstraße 14 UZA II, 1090 Vienna, Austria

**Keywords:** Mediterranean diet, Cancer, Dietary pattern, Systematic review

## Abstract

Overall cancer incidence has been observed to be lower in Mediterranean countries compared to that in Northern countries, such as the UK, and the USA. There is increasing evidence that adherence to a Mediterranean dietary pattern correlates with reduced risk of several cancer types and cancer mortality. In addition, specific aspects of the Mediterranean diet, such as high consumption of fruit and vegetables, whole grains, and low processed meat intake, are inversely associated with risk of tumor pathogenesis at different cancer sites. The purpose of this review is to summarize the available evidence regarding the association between the Mediterranean diet and cancer risk from clinical trials, prospective cohort studies, and case–control studies. Furthermore, we focused on the different definitions of a Mediterranean diet in an attempt to assess their efficiency. Observational studies provide new evidence suggesting that high adherence to a Mediterranean diet is associated with reduced risk of overall cancer mortality as well as a reduced risk of incidence of several cancer types (especially cancers of the colorectum, aerodigestive tract, breast, stomach, pancreas, prostate, liver, and head and neck).

## Introduction

In 2013, the number of deaths worldwide and throughout all age groups reached nearly 55 million, with 70 % of them caused by non-communicable diseases, including 15 % caused by cancer [1]. In spite of a decrease in death due to neoplastic disease since 1990, cancer remains a tremendous health problem. According to estimations by the International Agency for Research on Cancer, the 5-year global cancer prevalence is ∼28.8 million in 2008. With respect to localization, the most prevalent cancer worldwide continues to be carcinoma of the breast. Prostate cancer is the most common neoplastic disease in the USA and Oceania as well as Western and Northern Europe [2].

For several decades now, observational studies point out that the incidence of overall cancer is lower in countries bordering the Mediterranean Sea when compared to that in Northern countries, the UK, and the USA [3]. The concept of a “Mediterranean diet” (MD) was developed to reflect the typical dietary habits adopted during the early 1960s by inhabitants of the Mediterranean basin, mainly in Crete, much of the rest of Greece, and Southern Italy [4]. Adherence to a MD has previously been reported to be effective in the primary and secondary prevention of a number of chronic non-communicable diseases, such as cardiovascular diseases [5•], neurodegenerative diseases [6], type 2 diabetes mellitus [7], and neoplastic diseases [8, 9].

In 2014, we published a meta-analysis of observational studies investigating the effects of conformity with an MD on overall cancer risk (incidence and mortality) and risk of development of different types of cancer [10••]. Our findings further underlined the importance of a MD as a potential health-promoting dietary pattern, and it became reasonable to update this systematic review only a year later due to the large number of additional observational studies (14 cohort and 9 case–control studies) published in the interim brief period [11••]. In both meta-analyses, adherence to the highest category of MD was associated with a significant reduction in the risk of overall cancer mortality/incidence as well as the incidence of several cancer types (especially cancers of the colorectum, aerodigestive tract, breast, stomach, pancreas, prostate, liver, and head and neck).

The number of cancer survivors in the USA and Europe is rapidly growing [12, 13]. A few prospective cohort studies investigated the association between composition of the diet and cancer survival, reporting inconsistent results [14•]. For example, several studies focused on the evaluation of the relationship between survival and nutrients rather than dietary patterns [14•, 15]. The objective of the current review was to summarize the available evidence on Mediterranean diet and cancer risk (with respect to incidence, survival, and mortality) as well as to report on current results and futures directions. Prior to that, we provide an overview of the most common ways to define a Mediterranean diet.

## What is a Mediterranean Diet?

The most commonly used operational definition of a MD is the Mediterranean Dietary score proposed by Trichopoulou et al. in 1995 [16], which was updated in 2003 [17]. The Mediterranean score is built by assigning a value of 0 or 1 to each of nine components with the use of sex-specific medians as the respective cutoffs. In detail, this MD score is characterized [17] by six beneficial components (high consumption of vegetables, fruits and nuts, legumes, unprocessed cereals, fish, and a high ratio of monounsaturated fatty acids to saturated fatty acids) and two components regarded to be detrimental (meat and meat products including poultry and dairy products with the exception of cheeses preservable for a long period of time). With respect to favorable ingredients, persons whose consumption is at or above the median receive a score of 1 for each category, and persons whose consumption is below the median receive a score of 0. With respect to unfavorable ingredients, the scoring system is applied with reversed signification. The potential benefit of moderate alcohol consumption is taken into account by assigning a value of 1 to men who consume between 10 and 50 g per day and to women who consume between 5 and 25 g per day, respectively [17]. The highest adherence to a MD is therefore represented by a maximum score of 9.

The second most widely used MD score is an alteration of the original score by Trichopoulou adapted by Fung et al. in 2006 [18]. The following components were either excluded or modified: exclusion of potato products from the vegetable group, separation of fruits and nuts into two groups, exclusion of the dairy group, inclusion of whole-grain products only, inclusion of red and processed meats only in the meat group, and assignment of 1 point for alcohol intake between 5 and 15 g/day. In 2014, Whalen et al. [19] modified this alternate MD score with respect to dairy foods, grains and starches, and alcohol intakes. Another variation of the score by Trichopoulou et al. [17] was introduced by Tognon et al. [20] and Xie et al. [21] by adding fruit juices and polyunsaturated fatty acids, focusing on whole grains, and excluding poultry. The MD score established by Panagiotakos et al. in 2007 [22, 23] is based on 11 main components (non-refined cereals, fruits, vegetables, potatoes, legumes, olive oil, fish, red meat, poultry, full-fat dairy products, and alcohol). Consumption of items adhering to this pattern will result in scores of 0, 1, 2, 3, 4, and 5 when a participant reported no, rare, frequent, very frequent, weekly, and daily consumption, respectively. Three of the 11 components were considered to exert detrimental effects (red meat, poultry, full-fat dairy products). For alcohol, a value of 5 is assigned to subjects who consume <300 ml (12 g ethanol) alcoholic beverages per day, and a value of 0 is applied if alcohol consumption is either >700 or 0 ml/day.

Other infrequently used MD scores include the Mediterranean Diet quality index [24], the Mediterranean Score by Goulet et al. [25], the Mediterranean Diet pattern score [26], the Mediterranean dietary pattern adherence index [27], and the Mediterranean Adequacy Index [28].

## Mediterranean Diet and Cancer Risk

### Overall Cancer Mortality

#### RCTs

The first clinical trial to demonstrate the protective effects of the MD in the secondary prevention of ischemic heart disease was the Lyon Diet Heart study. Participants were asked to replace butter and cream with a special alpha-linolenic-acid-rich margarine. Apart from cardiovascular diseases, protective effects of an MD were also reported with respect to the risk of cancer development [29]. Results of the study were transferred into the following food advices: to increase the consumption of vegetables, fruits, and fish, but to reduce the consumption of red meats.

Besides the Lyon Diet Heart study, there is only one other randomized controlled trial of a Mediterranean diet reporting on cancer risk. The “Prevención con Dieta Mediterránea” (PREDIMED) trial focused on the intake of extra virgin olive oil (50 g/day) and nuts (30 g/day) in the intervention groups [30••]. Results including 7447 subjects showed that the highest category of nut intake (>3 servings/week) was associated with a 40 % risk reduction of cancer mortality when compared to the lowest category [31•], whereas the differences observed between consumption categories of extra virgin olive oil did not attain statistical significance [32•].

#### Prospective Cohort Studies

Overall risk of cancer mortality was evaluated in 11 cohort studies (European Prospective Investigation into Cancer and Nutrition (EPIC), Nurses’ Health Study, Health Professionals Follow-up Study, Aerobics Center Longitudinal Study, Women’s Health Initiative Observational Study, Multiethnic Cohort, HALE and SENECA study, SUN-cohort, Seven Countries Study, National Institutes of Health American Association of Retired Persons (NIH-AARP), Västerbotten Intervention Program-cohort, Swiss National Research Program 1A, MONICA) [20, 33–42]. Six of these cohorts did not show a significant correlation between adherence to an MD and cancer risk. However, pooling all 11 cohort studies in a meta-analysis yielded a 13 % risk reduction of overall cancer mortality when comparing the highest versus lowest adherence to MD categories [11••].

## Cancer Localizations

### Breast Cancer

Five prospective cohort studies [18, 20, 43–45] and eight case–control studies [46–53] investigated the effects of conformity to an MD and risk of breast cancer. Pooling eight case–control studies resulted in a 10 % reduction of breast cancer incidence. However, pooling cohort studies (which are characterized by a higher level of evidence) did not confirm these results, i.e., none of the prospective cohort studies showed a significant inverse association between concordance to MD and breast cancer risk. Furthermore, we detected a trend for an association between high adherence to MD and reduced risk of breast cancer in postmenopausal women. Differentiating breast cancer types as classified by receptor status yielded significant results when comparing the highest versus lowest adherence category to MD only for the ER−/PR+ type (relative risk (RR) 0.71, 95 % confidence interval (CI) 0.56 to 0.89) [11••].

### Colorectal Cancer

Four prospective cohort studies [20, 54–56] and four case–control studies [19, 57–59] investigated the effects of adherence to MD and risk of colorectal cancer. Of these eight studies, two cohort and three case–control studies demonstrated a significant inverse association between adherence to MD and incidence of colorectal cancer. Following synthesis of all results via meta-analysis (excluding the study Tognon et al. 2012 on colorectal cancer mortality), a 17 % risk reduction for colorectal cancer could be detected when juxtaposing the highest versus lowest MD categories [11••].

### Prostate Cancer

The risk of prostate cancer could be reduced by 4 % comparing the highest versus lowest adherence to MD category including four cohort studies [20, 60–62] and one case–control study [63].

### Gastric Cancer

The EPIC study demonstrated a 33 % reduced risk of gastric adenocarcinoma following comparison of the third versus the first tertile of conformity to an MD [64]. Pooling all available cohort [64, 65•] and case–control [66] studies revealed a 27 % reduced risk of gastric cancer incidence [10••].

### Liver Cancer

To date, only two studies (one cohort [67] and one case–control study [68]) investigated the effects of MD adherence on liver cancer risk. Both studies observed a significant inverse association (42 % reduced risk) [11••]

### Esophageal Cancer

The NIH-AARP-cohort study including nearly 500,000 subjects showed a significant risk reduction for esophageal squamous cell carcinoma (RR 0.44, 95 % CI 0.22 to 0.88), but not for esophageal adenocarcinoma [65•] in individuals adopting a MD pattern. An Italian case–control study reported a lower odds ratio for esophageal cancer in participants with high adherence to MD [69].

### Head and Neck Cancer

Pooling one cohort study [70] and three case–control studies [69, 71, 72] in a meta-analysis resulted in a significantly reduced risk of head and neck cancer incidence (RR 0.40, 95 % CI 0.24 to 0.66) when comparing the highest versus the lowest categories of MD adherence. However, the data turned out to be non-significant in a sensitivity analysis considering only data from cohort studies [11••].

### Pancreatic Cancer

Both a Swedish cohort study (pancreatic cancer mortality) [20] and an Italian case–control study [73] reported a significant inverse association between the highest concordance category to MD and pancreatic cancer risk. However, pooling these two studies resulted in a non-significant risk reduction, probably due to high between-study heterogeneity.

### Ovarian, Bladder, and Endometrial Cancer

No significant association between adherence to Mediterranean diet and risk of ovarian cancer could be observed in the Nurses’ Health Study [21]. Including 477,312 subjects from the EPIC study indicated a negative association between adherence to a MD pattern and risk of bladder cancer that was, however, non-significant [74]. Among postmenopausal women in the Women’s Health Initiative Clinical Trial and Observational Study, high conformity to MD (fifth vs. first quintile) was not significantly associated with risk of endometrial cancer [75]. In contrast, data from three case–control studies showed that high adherence (six to nine components) to MD correlated with a reduced odds ratio (OR 0.43) for endometrial cancer incidence [76], while a fourth case–control study from the USA did not confirm these protective effects of a MD [77].

### Respiratory Tract Cancer

Two cohort studies investigated the effects of adherence to MD and risk of respiratory tract cancer [20, 78]. Pooling their data yielded a non-significant correlation between MD and reduction of respiratory tract cancer incidence [11••].

## Mediterranean Dietary Pattern and Cancer Survivors/Recurrence

The potential correlation between conformity to a high MD score and cancer mortality among cancer survivors was evaluated in three cohort studies [62, 79•, 80] while cancer recurrence among cancer survivors was assessed in one cohort study [81]. No significant association between MD and cancer mortality or cancer recurrence could be observed.

## Diet Among Cancer Survivors

Tumor progression and recurrence as well as survival of cancer patients were shown to be affected by aspects of nutrition such as macronutrient composition or supplementation with specific nutrients, and some of these research studies have been transferred into recommendations and guidelines for cancer survivors [13, 14•]. Moreover, there are some studies dealing with the effects of food groups in cancer patients. A diet emphasizing fruits and vegetables, whole grains, and fish, but low amounts of red or processed meat and sugars, was associated with decreased mortality rates in cancer survivors [82–84]. Taken together, this description fits well with the characteristics of a Mediterranean diet. In the last part of this review, we will take a closer look at some of these components and their potential interactions with cancer development and progression focusing on data provided by systematic reviews and meta-analyses.

## Components of Mediterranean Diet and Cancer

### Fruit and Vegetables

A protective effect of fruits and vegetables on cancer initiation and progression might be explained by their high content of flavonoids exerting favorable biological effects such as anti-oxidant, anti-inflammatory, anti-mutagenic, or anti-proliferative properties [85]. Prospective cohort studies yielded inconsistent results regarding fruit and vegetable intake and cancer risk. Data from the EPIC cohorts provide evidence of at least a weak inverse association between high consumers and lower risk [86, 87]. However, these findings are not uniform as shown, e.g., by George et al. [88] following analysis of data from the cohort of the National Institutes of Health-AARP Diet and Health Study. In a recent meta-analysis synthesizing data from 16 prospective cohort studies, a higher consumption of fruit and vegetables did correlate with a decrease in all-cause mortality, albeit not with a significant reduction in cancer-related death [85].

### Fish

A higher consumption of fish is recommended especially due to its content of n-3 fatty acids. Via their anti-inflammatory effects, n-3 may suppress carcinogenesis and act in a protective manner against tumor initiation and progression. However, recent meta-analyses show rather inconsistent and inconclusive results with respect to inverse relationships between uptake of marine n-3 fatty acids or fish consumption and cancer incidence/mortality [89•, 90].

### Whole Grain

Increased uptake of dietary fiber is regarded to be protective especially against the development of colorectal cancer, e.g., via an enlargement of the bulk of stool leading to a concomitant reduction in transit time thereby diminishing the impact of potential carcinogenic substances on the colonic epithelium. In addition, metabolization of fiber to short-chain fatty acids by intestinal bacteria may prevent dedifferentiation of colonic cells and support defense mechanisms against initiated cells by apoptosis [91]. In fact, there are a number of data from observational studies consistent with this hypothesis. Thus, consumption of whole grains and dietary fiber was inversely associated with risk of developing colorectal cancer in meta-analyses by Haas et al. [92] as well as Aune et al. [93]. Moreover, intervention and observational studies showed that dietary fiber is associated with reduced risk of insulin resistance [94, 95].

### Olive Oil

Systematic analyses of epidemiological studies on the effects of olive oil on cancer development indicated decreased relative risks or odds ratios for breast cancer, cancers of the digestive system, or neoplasms of the respiratory tract [92]. Extra-virgin olive oil is rich in both monounsaturated fatty acids and in polyphenolic compounds such as tyrosol, hydroxytyrosol, and oleuropein. It has been speculated that the phenolic content of extra virgin and virgin olive oil is able to specifically affect cancer-regulated oncogenes [96–98]. Furthermore, they may exert strong chemo-preventive effects via a variety of distinct mechanisms, including both direct anti-oxidant effects and actions on cancer cell signaling and cell cycle progression [99].

### Alcohol and Red Wine in Moderate Amounts

Moderate consumption of alcohol in a MD is usually characterized by a threshold of <30 g ethanol/day for men and less than 20 g/day for women with a focus on red wine containing secondary plant substances such as polyphenols. These light amounts of alcohol were found to be associated with a reduced risk of cardiovascular disease in a number of observational studies. On the other hand, alcoholic beverages providing ≥30 g ethanol/day are associated with increased risks of different cancers (e.g., cancers of the pharynx, larynx, esophagus, colorectum, breast, and liver) [100]. The effects of light alcohol drinking on cancer development and progression are still discussed controversially. While moderate consumption of less than 12.5 g ethanol/day was found to correlate with a decreased risk of cancer mortality when compared to non-drinkers [101•], the same amount of alcohol was reported to increase the risk of cancer for various locations in a meta-analysis by Bagnardi et al. [102].

### Red and Processed Meat

Within the context of a MD, red and processed meat (and sometimes poultry) are regarded to be unfavorable compounds which should be consumed in low amounts. There is considerable evidence derived from meta-analyses linking high consumption of meat and/or processed meat with an increased risk of cancer mortality [103]. In addition, a meta-analysis of 21 observational studies presented by Xu et al. demonstrated that high consumption of red meat/processed meat is associated with an increased risk of colorectal adenomas [104].

### Dairy Products

Different and sometimes contradictory results are reported with respect to consumption of milk and dairy products and the risk of cancer. In a recent report of two independent Swedish cohort studies, Michaelsson and co-workers [105] could demonstrate an increased adjusted hazard ratio for each glass of milk with respect to death caused by cancer in a female cohort. On the other hand, meta-analyzing the data available from cohort studies does not confirm a detrimental effect of dairy products. In contrast, milk and dairy product consumption was associated with a decreased relative risk for development of colorectal cancer [106], and total dairy food intake was found to be associated with a decreased risk of breast cancer [107].

## Conclusion

It was the aim of the present review to summarize evidence provided by randomized and observational studies on the potential effects of a Mediterranean diet on cancer development, progression, and mortality. The data support the concept that a MD has a benefit with respect to these clinical outcomes, i.e., adherence to a MD was associated with reduced risk of overall cancer mortality as well as incidence of colorectal, breast, gastric, prostate, liver, and head and neck cancer. When dismantling the Mediterranean diet into its components, it seems that there is no single ingredient or food category mediating these favorable effects. It seems instead to be the result of the complex food pattern characteristic for a MD. However, one should keep in mind the limitations of and differences between the underlying studies. Apart from the problems stemming from the design of observational studies in general, a strong methodological issue is the wide variety of scores used to identify adherence to a MD. Nevertheless, taking into account the increasing number of observations reporting a beneficial effect of a Mediterranean diet in the primary and secondary prevention of other chronic diseases as well, it does not seem reasonable to exclude an MD pattern from dietary recommendations aimed at cancer prevention.
